# Emerging Role of Immunity in Cerebral Small Vessel Disease

**DOI:** 10.3389/fimmu.2018.00067

**Published:** 2018-01-25

**Authors:** Ying Fu, Yaping Yan

**Affiliations:** ^1^Key Laboratory of the Ministry of Education for Medicinal Resources and Natural Pharmaceutical Chemistry, National Engineering Laboratory for Resource Development of Endangered Crude Drugs in Northwest of China, College of Life Sciences, Shaanxi Normal University, Xi’an, China

**Keywords:** cerebral small vessel disease, degeneration, pathogenesis, inflammation, autoimmune response

## Abstract

Cerebral small vessel disease (CSVD) is one of the main causes of vascular dementia in older individuals. Apart from risk containment, efforts to prevent or treat CSVD are ineffective due to the unknown pathogenesis of the disease. CSVD, a subtype of stroke, is characterized by recurrent strokes and neurodegeneration. Blood–brain barrier (BBB) impairment, chronic inflammatory responses, and leukocyte infiltration are classical pathological features of CSVD. Understanding how BBB disruption instigates inflammatory and degenerative processes may be informative for CSVD therapy. Antigens derived from the brain are found in the peripheral blood of lacunar stroke patients, and antibodies and sensitized T cells against brain antigens are also detected in patients with leukoaraiosis. These findings suggest that antigen-specific immune responses could occur in CSVD. This review describes the neurovascular unit features of CSVD, the immune responses to specific neuronal and glial processes that may be involved in a distinct mechanism of CSVD, and the current evidence of the association between mechanisms of inflammation and interventions in CSVD. We suggest that autoimmune activity should be assessed in future studies; this knowledge would benefit the development of effective therapeutic interventions in CSVD.

## Introduction

Cerebral small vessel disease (CSVD) represents a diverse range of pathological changes that affect capillaries, small arteries and small veins in the brain. This disease is related to lacunar infarct, microbleeds, enlarged perivascular spaces, leukoaraiosis, and cortical atrophy. As such, CSVD causes 20% of strokes and constitutes a main source of cognitive decline, particularly in the elderly (1–4). However, apart from risk containment, efforts to prevent or to treat CSVD are ineffective (5, 6). The burdens of dementia and the cost to society imposed by CSVD are overwhelming and have incited efforts to explore new therapeutic resources (7, 8).

Immune responses have recently emerged as important elements contributing to the progression of stroke. Recent reviews in the literature have discussed the contribution of inflammatory mediators and lymphocytes to the development of brain lesions and neurological deficits that occur in acute ischemic stroke with large artery occlusion or acute cerebral hemorrhage (9–15). Recurrent minor stroke attacks in CSVD lead to blood–brain barrier (BBB) leakage (16–19), central nervous system (CNS) antigen release into the peripheral circulation and lymphocyte infiltration into brain tissue, which allow for the possibility of novel antigens deprived from the CNS to encounter the lymphocytes (20, 21). In addition to BBB disruption, blood proteins at the neurovascular unit activate microglia to produce chemokines, which cause peripheral inflammatory cells to migrate to the CNS, create a chronic inflammatory microenvironment and encourage activated lymphocytes to encounter CNS antigens (22–28). Immune responses in CSVD are not well characterized and may contribute to the pathogenesis of CSVD injury just as to those of multiple sclerosis (MS) and neuromyelitis optica (NMO), classic autoimmune disorders of the CNS. Therefore, we will focus on identifying specific characteristics of the role of the immune system in CSVD. We will compare imaging, pathology and immune features with MS. Such comparisons will be considered in relation to the use of disease-modifying drugs and their abilities to control the progression of CSVD. We believe that the identification of the differences and similarities in the immune mechanisms involved in CSVD and MS may potentially provide valuable hints to harness the use of disease-modifying drugs for the attenuation of inflammation and to improve clinical outcomes of patients with CSVD just as those in MS. The results from proof-of-concept clinical trials with fingolimod in both acute ischemic stroke and intracerebral hemorrhage (29–32), together with natalizumab in acute ischemic stroke (33), suggest that this concept is not only reasonable but also feasible (33).

## CSVD and Stroke

Stroke comprises the following pathological types: intracerebral hemorrhage, subarachnoid hemorrhage and ischemic stroke. Lacunar-type strokes account for 20–30% of ischemic strokes (34). Moreover, small hemorrhages and microbleeds can occur in lacunar stroke (35). Although lacunas and small hemorrhages may appear after clinical attacks, most of these types of stroke develop “silently.” Experiencing numerous strokes is associated with diffuse white matter hyperintensities, cerebral atrophy, and enlarged perivascular space and thus doubles the risk of dementia (1, 36, 37). This triggering of both small ischemic and hemorrhagic consequences by pathological small vessels and cerebral degeneration is collectively known as CSVD (4).

## Blood Proteins at the Neurovascular Unit Promote Immune Action in the Brain

Fibrin is a result of thrombin-mediated conversion of fibrinogen to an insoluble fibrin network, as the final product of the coagulation cascade. Human studies and experimental animal models provided evidence for the critical role of fibrin in inflammation (38, 39). Interactions between fibrin and microglia *via* TLR4 and CD11b/CD18 receptors were identified as direct activation pathways of the innate immune response (23, 40). Fibrin-induced activation of microglia triggers chemokine and cytokine secretion and stimulates leukocyte recruitment, thus leading to an inflammatory environment in the neurovascular unit (39). Importantly, Ryu et al. found that fibrin in the neurovascular unit of MS models was sufficient to induce the activation of myelin-specific T cells and infiltration into the CNS, demonstrating that a fibrin-induced innate immune response triggers CNS autoimmunity (23, 40). Under normal conditions, blood proteins such as plasmin and fibrinogen are not detected in the parenchyma of the brain shielded by the intact BBB. In response to BBB disruption and components from the blood entering the brain milieu, blood proteins-associated inflammation occurs in the CNS parenchyma.

Cerebral small vessel disease models, including chronic cerebral hypoperfusion and spontaneously hypertensive rats, have identified deficits in BBB integrity, which suggests a close spatial and temporal relationship between the extravasation of plasma constituents, brain tissue injury and subsequent inflammatory processes (41–45). BBB permeability has also been reported in CSVD patients. Albumin increases in the cerebrospinal fluid (CSF) of stroke patients (46, 47). Intrinsic small vessel disease results in vessel wall thickening, focal arteriolar dilatation, striking loss of normal vessel wall architecture, and extravasation of blood components into and through the wall; these findings were observed in post-mortem examinations (48–50). Neuroimaging provides considerable insights into the earliest stages of CSVD. Imaging studies revealed that BBB leakage is very subtle, persistent, and more spatially extensive in patients with CSVD (16, 18, 19); it even occurs prior to development of brain lesions (19).

Inflammatory cell infiltrations in the arteriolar wall and perivascular tissue have been noted in CSVD patients since 1902 (51–53). Moreover, clinical pathological data also demonstrated that the activation and proliferation of microglia induced the expression of MHC II and costimulatory molecules CD40 and B7-2, and the appearance of these cells in the parenchyma was accompanied by the disruption of the BBB and fibrinogen deposition, indicating that immune activation results from BBB disruption (54, 55). However, the mechanism of immune cell infiltration and activation is poorly understood in CSVD. More importantly, the contribution of immune cells to the development and progression of CSVD is also unclear.

A number of experimental studies were conducted to reveal the inflammatory pathogenesis mechanisms in CSVD (21, 56). Rosenberg et al. found that BBB disruption and MMP-9-mediated migration of T lymphocytes was related to extensive white matter abnormalities and behavioral impairments in chronically hypertensive rats. Minocycline, which has anti-inflammatory actions, including MMP-9 inhibition, effectively restored white matter integrity in SHR-SP (45). Weise et al. also showed that SHR-SP developed brain atrophy, white matter loss, BBB leakage, microglial activation with IL-1β secretion, and lymphocyte migration, suggesting a role for NK and T cells in cerebrovascular inflammation and hypertension-related cognitive decline (21).

## Immunity in Stroke

Acute insults to the brain in cerebral ischemic stroke or cerebral hemorrhage cause neuronal cell death and elicit local and diffuse inflammation. Damage-associated molecular patterns trigger resident cells and initiate cellular and humoral cascades (57, 58). Such inflammatory cascades induce the overexpression of adhesion molecules and increase BBB permeability, thus favoring cumulative inflammatory cell infiltration and contributing to an increase in local and global brain damage (13, 14, 59). Furthermore, the continuous cytokine release starts a chronic inflammatory process that allows the dynamic shift of the macrophage and microglial canonical phenotype between M1 (classical activation) and M2 (alternative activation that is presumably the result of antigen-presenting cells migrating from the periphery) (10, 60).

The presence of autoimmune responses to brain antigens in stroke patients has been reported since the early 1970s (61–64). Shortly after stroke onset, brain-derived antigens (e.g., MBP, GFAP, CK-BB, NSE, and S100) were present within the peripheral circulation (65, 66) and cervical lymph nodes (67, 68). In addition, lymphocytes traffic into the infarcted brain tissue within days after stroke (69–72), allowing for the possibility of a novel antigen to encounter the CNS (7). Concerning the systemic immune system, these antigens are essentially novel, indicating that lymphocytes encountering such an antigen could lead to the development of an autoimmune response (6).

In recent years, Becker et al. conducted a series of studies about autoimmunity in stroke, mainly the cellular immune response. Similar to other clinical studies, they found that cellular immune responses (Th1 type) to brain antigens occurred in patients with acute stroke (73–76). Furthermore, they found that the Th1 response to MBP was an independent predictor of stroke outcome, and more robust cellular responses to MBP were associated with a decreased likelihood of a good outcome (76). The same results were also found in stroke models (77, 78). At the time of stroke, animal models subjected to infections or systemic inflammatory stimuli are predisposed to develop an autoimmune response to the brain, and this response is related to poor outcomes (79–81). Accordingly, the induction of MBP-induced or MOG-induced tolerance was found to prevent CNS autoimmunity and improve outcomes in experimental stroke (82–86). Offner et al. also found that MOG-reactive cells invaded the CNS and exacerbated stoke severity, further substantiating the idea that the cellular immune response might affect stroke outcomes (87). In contrast, Meisel et al. showed that stroke-induced immunodepression might represent an adaptive mechanism that inhibited long-lasting antigen-derived brain cellular immune responses (88, 89).

The presence of antibodies to brain antigens has been described in stroke. Immunoglobulins are present in the CSF of approximately 25% of survivors in the chronic phase of stroke (90–92). Some autoantibodies to brain antigens (e.g., MBP, PLP, NF, and NR2A/2B) have been documented in individuals after stroke (93–97). In a study with 40 patients, anti-MBP antibody titers were associated with cognitive decline during the first year after stroke (98), but we still do not completely understand the pathological consequences of this humoral response. In a stroke model study, researchers found that mice with B lymphocyte infiltrates in their infarct cores developed late cognitive decline and that blocking the B cell response using a mouse analog of rituximab, an FDA-approved anti-CD20 antibody, prevented this cognitive decline. This result provides evidence that autoantibodies can interfere with neuronal function and could mediate cognitive impairment after stroke (99).

**Figure 1:**
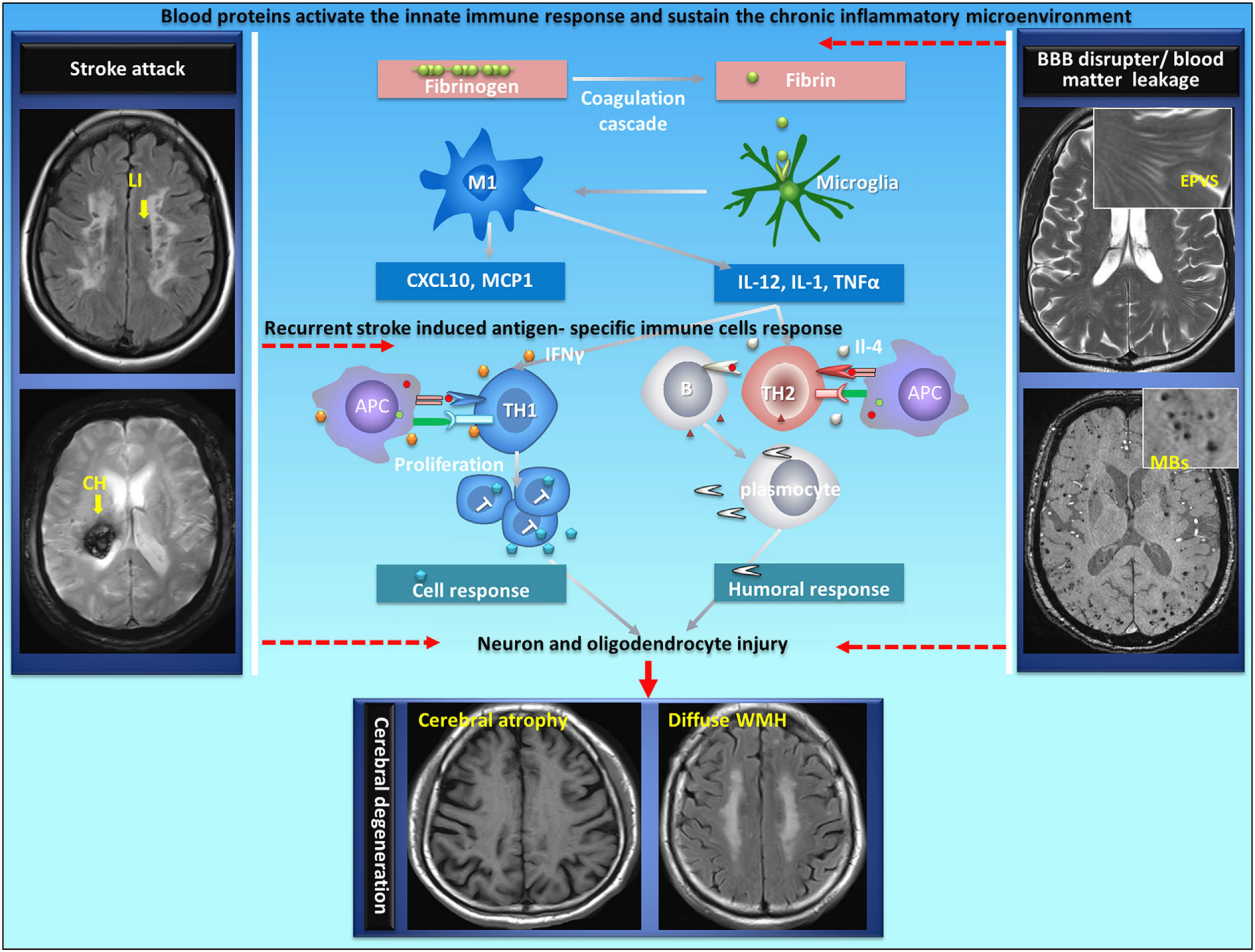
Proposed autoimmunity mechanism in the development of neurodegeneration in CSVD. CSVD is a cerebral vascular disorder characterized by recurrent strokes with sustainable BBB disruption as well as a chronic inflammatory response at the neurovascular unit. Autoimmunity could be generated in acute stroke under certain brain chronic inflammatory circumstances with damaged immune tolerance in CSVD. Blood proteins at the neurovascular unit play an important role in the communication between the brain and the immune system. During BBB disruption, fibrinogen extravagates into the CNS and is converted to fibrin upon activation of coagulation. Fibrin, the high-affinity plasma-derived ligand for CD11b/CD18, activates CNS-resident innate immune cells (microglia and perivascular macrophages) to stimulate cytokine release, thus sustaining antigen-presenting properties by providing instructive signals (such asIL-12, IL-1, and TNFα) to promote antigen-specific (neuron or oligodendrocyte) Th1-cell or Th2-cell differentiation following a stroke. The cellular immune response or humoral immune response leads to neuron and oligodendrocyte injury. APC, antigen-presenting cells; LI, lacunar infarct; CH, cerebral hemorrhage; EPVS, enlarged perivascular space; MBs, microbleeds; CSVD, cerebral small vessel disease; BBB, blood–brain barrier. The original data were acquired in the YPY group.

The type of immune response that develops to a particular antigen is dependent upon the microenvironment at the site of antigen encounter (100). Th1-type response, which is associated with the cellular immune response, is favored by an inflammatory microenvironment where IFN-γ is present, such as what might occur during a systemic infection; Th2-type response, which is classically associated with humoral immunity and antibody secretion, is favored by the presence of cytokines such as IL-4 (101–105). However, the cellular immune response or humoral immune response depends on the local microenvironment and the presence of costimulatory molecules. CSVD is a cerebral vascular disorder characterized by recurrent strokes with sustainable BBB disruption as well as a chronic inflammatory response at the neurovascular unit. Therefore, it is possible that immune tolerance could be damaged in stroke under certain chronic inflammatory circumstances in CSVD. As mentioned previously, blood proteins at the neurovascular unit play an important role in the communication between the brain and the immune system (Figure 1). However, it is still unknown whether fibrin triggers and sustains antigen-specific lymphocytes in the CNS of patients with acute brain injury in chronic phase.

## Understanding the Unique Immune Mechanisms in CSVD is Instrumental for Immune Interventional Therapies

Stroke does not systematically trigger autoimmunity; however, under certain circumstances such as pronounced microenvironment inflammation, autoreactive T cells could escape the tolerance controls and induce antigen-specific immune responses (Figure 1). CSVD is characterized by recurrent strokes with cumulative disabilities and vascular dementia (Table 1). At the onset of ischemic and hemorrhagic stroke (attack phase), emerging evidence has revealed that stroke induced a local inflammatory reaction and a plethora of innate immune responses in the brain where antigen-presenting cells became prominent; following the onset of stroke, inflammatory components (IL-4 or IFN-γ), which are produced by innate immune cells (e.g., microglia, NK cell) with the stimulation of blood proteins at the neurovascular unite, promote detrimental cellular or humoral responses and lead to diffuse neuron and oligodendrocyte damage (101–105). In chronic stages (remitting phase), the chronic inflammatory activity that is triggered by blood proteins at neurovascular units might also participate in post-stroke cognitive decline and neurodegeneration (39, 40).

**Table 1:** Contrasting features of clinical, imaging, pathology and inflammation between CSVD and MS.

	CSVD	MS
**Clinical features**		
Course of disease	A chronic disease	A chronic disease
Attack events	Lacunar infarct and cerebral hemorrhage	Inflammatory demyelination activation
Disability	Accumulation	Accumulation
Neurodegeneration	Cognition, gait, neuropsychology and sleep disturbance	Cognition, gait, neuropsychology and sleep disturbance

**Brain MRI**		
T2/FLAIR white matter hyperintensities	Focal and diffuse	Focal and diffuse
T1 hypointensities	Transient and persistent	Transient and persistent
Microbleeds	Common	Rare
Contrast enhancing lesions	Common at stroke recurrent stage, rare at remitting stage	Common at relapse phase, rare at remitting stage
Enlarged perivascular space	Centrum semiovale and basal ganglion region	Centrum semiovale region
Cerebral atrophy	Gray matter reduced and ventricles gradually expanded	Gray matter reduced and ventricles gradually expanded

**Pathology features**		
Demyelinating region	Arterial watershed areas	High venule density and arterial watershed areas
Myelin	Selective loss of phospholipids and MAG with PLP preservation	Myelin loss with selective reduction of phospholipids
Axonal	Loss	Loss
Blood–brain barrier	Increased permeability and fibrin leakage	Increased permeability and fibrin leakage
Perivascular	Perivascular collagenases and inflammatory	Perivascular collagenases and inflammatory cuffs
Inflammatory cell	Microglia and astrocyte activation and lymphocytic infiltration	Microglia and astrocyte activation and lymphocytic infiltration

**Inflammation features**		
Triggering events for immune activation	Cell death products, microglia activation	Mostly unidentified
Location of activation signals	Brain and periphery	Periphery
Antigen specificity	Mostly antigen-specific cells and antigen-specific antibody	Mostly antigen-specific cells
Immune effector cells	Combined effects of many cells, no dominant cell type	Coordinated events dominated by T cells
Role of inflammatory mediators	Presumably many, including IFN-γ, IL-17, IL-4	Presumably many, including TNF-a, IFN-γ, IL-17
Efficacy of immune modulation	Under investigation	13 FDA-approved, disease-modifying drugs, moderate to high efficacy

The slow developments of CSVD suggest that exploring the mechanisms and interventions for its prevention or treatment will need long-term study for recurrent acute minor stroke and chronic progress neurodegeneration. A disease-modifying strategy aimed at changing the natural course of an illness is primarily applied to treat chronic diseases. In the field of neurological disorders, this concept has been used for neuroinflammatory diseases such as MS. Given the similarities in the inflammatory mechanisms and clinical characters of MS and CSVD (Table 1), one would ideally expect that CSVD requires a similar immunotherapeutic and preventive approach to that used for MS.

Fingolimod became the first oral drug to be FDA-approved for the treatment of relapsing-remitting MS. This drug can act on four of the five known S1P receptor subtypes (S1PR1, S1PR3, S1PR4, S1PR5) and exerts its immunomodulatory actions by affecting lymphocyte numbers, trafficking and apoptosis through S1P receptors. Specifically, fingolimod reduces circulating lymphocytes by preventing their egress from lymph nodes during stroke, and fingolimod might contribute to the prevention of the early infiltration of lymphocytes into the brain, thus reducing thromboinflammation (106–108). In our three open-label trials, patients with acute ischemic or hemorrhagic stroke were treated with oral fingolimod for 3 days after the onset of symptoms, and consequently, microvascular permeability and secondary injury were reduced in these patients (29–32). However, the action of fingolimod in acute stages involved in diffuse brain injury and cerebral degeneration is still poorly understood and needs to be elucidated (109, 110). A recent study found that fingolimod could induce VEGF expression of astrocytes by stimulating S1PR3, which plays a role in the breakdown of the BBB, a step critical to the entry of pathogenic lymphocytes into the brain (111). Of note, BBB leakage induced by fingolimod due to the activation of S1PR3 in astrocytes may limit its use, and selective S1PR1 agonist (e.g., LASW1238, RP101075) treatment should be further optimized (112, 113).

Natalizumab blocks α4-integrin, which mediates the invasion of lymphocytes (mainly T cells) into the CNS, and currently represents one of the most effective therapies for relapsing-remitting MS. The ACTION study, a randomized controlled phase IIa trial comparing the effect of a single injection of 300 mg of intravenous natalizumab and placebo within a 9-h time window after symptom onset, found no effect of natalizumab on infarct growth, but patients receiving natalizumab were more likely to have an excellent cognition outcome at 90 days. This outcome was particularly evident in subgroups of patients with smaller infarcts. This result suggests that mitigating diffuse neuroinflammation triggered by acute stroke may additionally mitigate cerebral degeneration, especially in minor stroke. Considering the safety and efficacy of fingolimod and natalizumab in acute stroke, future preclinical animal experiments and translational clinical trials involving fingolimod and natalizumab treatment for CSVD are expected.

Dimethyl fumarate (DMF) is utilized as an oral drug to treat MS and has been demonstrated to be as potent as several other drugs but with fewer side effects (114, 115). The beneficial effects of this medication were consistent with regulation of CD4^+^ Th1 cell differentiation. More importantly, DMF was discovered to impact the anti-oxidative stress cell machinery to promote the transcription of genes downstream of the activation of the nuclear factor Nrf2 (116, 117). It was reported that DMF might be useful for treating acute stroke. In acute stroke models, DMF prevented cerebral edema progression at the acute stage and promoted recovery at the chronic stage (118–120). Recently, an experiment using mice with bilateral common carotid artery stenosis revealed that DMF decreased microglia/macrophage activation, protected against white matter injury and improved cognition impairment (121). Multiple immunomodulatory and anti-oxidative stress actions support DMF as an appealing medication; however, its potential for impacting the degenerative aspects of CSVD remains to be explored.

Rituximab is FDA approved as a B-cell-depleting drug for rheumatoid arthritis, non-Hodgkin’s lymphoma, chronic lymphocytic leukemia, and microscopic polyangiitis. Rituximab was also found to be effective in decreasing the autoantigen-specific humoral immune response or inhibiting inflammatory responses orchestrated by pathogenic B cells in patients with MS and NMO (122–127). Although both deleterious and protective regulatory roles of B lymphocytes have been increasingly recognized, translation of these roles of B lymphocytes into clinical trials in stroke has not yet occurred. However, pharmacological ablation of B lymphocytes using rituximab after 5 days of stroke prevents the appearance of delayed cognitive deficits in an animal stroke model with large vessel occlusion (99). Nevertheless, this finding suggests that rituximab treatment could be a promising therapy for CSVD, given the production of brain-reactive antibodies associated with cognitive decline in stroke patients (98).

Minocycline is a tetracycline antibiotic agent that has multiple immune-modulating properties; clinical data have shown the activity of minocycline in patients with MS or clinically isolated syndrome with a good safety profile (128–132). Minocycline also reduces infarct size in acute stroke clinical trials (132, 133). More recently, Rosenberg et al. found that minocycline decreased hypoxia-induced infiltration of leukocytes, reduced white matter damage, improved behavior, and prolonged life in CSVD models (44, 45). Since minocycline is used as an antibiotic in the clinical setting, its safety for human use has been extensively evaluated. Moreover, the multiple neuroprotective effects of minocycline in vascular injury models support its use as a potential therapeutic treatment for CSVD (134–138).

## Conclusion and Future Directions

Brain proteins are detected in the blood of stroke/lacunar stroke patients (64, 66). Antibodies against brain antigens develop in patients with leukoaraiosis (94), suggesting a humoral immune response to the brain injury in CSVD. Furthermore, the presence of circulating T cells sensitized against brain antigens and antigen-presenting cells carrying brain antigens in the draining lymphoid tissue of stroke patients indicate that stroke might induce antigen-specific immune responses similar to those found in MS patients. We do not know whether poststroke dementia *via* lymphocyte-mediated autoimmunity has detrimental effects; however, clinical and preclinical trials of immune modulation using lymphocyte-targeted approaches have yielded some promising results in cognitive degeneration after stroke (33, 99). Impaired tissue oxygenation, induced inflammatory responses, and induced leukocyte infiltration are classical pathological features in CSVD (Table 1). In theory, mitigating chronic and diffuse neuroinflammation triggered by recurrent brain injury attack to prevent cerebral degeneration could be a feasible strategy against CSVD. However, one challenge to the advancement of the field is the incomplete understanding of the complex interactions between the immune system and the brain in CSVD. Therefore, the involvement of autoimmunity in CSVD should be cautiously assessed in future studies to facilitate the development of effective therapeutic interventions for CSVD.

## Author Contributions

YF and YY wrote and approved the final version of this manuscript.

## Conflict of Interest Statement

The authors declare that the research was conducted in the absence of any commercial or financial relationships that could be construed as a potential conflict of interest.
